# Identification of unhealthy alcohol use by self-report and phosphatidylethanol (PEth) blood concentrations in an acute psychiatric department

**DOI:** 10.1186/s12888-022-03934-y

**Published:** 2022-04-21

**Authors:** Trine Finanger, Arne Einar Vaaler, Olav Spigset, Trond Oskar Aamo, Trine Naalsund Andreassen, Rolf Wilhelm Gråwe, Ragnhild Bergene Skråstad

**Affiliations:** 1 Clinic of Substance Use and Addiction Medicine, St. Olav University Hospital, Klostergata 48, 7030 Trondheim, Norway; 2 Department of Clinical and Molecular Medicine, Faculty of Medicine and Health Sciences, Norwegian University of Science and Technology – NTNU, Trondheim, Norway; 3 Department of Acute Psychiatry, Division of Mental Health, St. Olav University Hospital, Trondheim, Norway; 4 Department of Mental Health, Faculty of Medicine and Health Sciences, Norwegian University of Science and Technology – NTNU, Trondheim, Norway; 5 Department of Clinical Pharmacology, St. Olav University Hospital, Trondheim, Norway; 6 Department of Research and Development, Division of Mental Health, St. Olav University Hospital, Trondheim, Norway

**Keywords:** Phosphatidylethanol, Mental health, Psychiatry, Alcohol use disorder

## Abstract

**Background:**

The use of standard screening methods could improve the detection rate of unhealthy alcohol use in patients admitted to psychiatric acute and emergency departments. The aim of the present study was to investigate the ability of the alcohol biomarker phosphatidylethanol (PEth) to identify patients with high levels of alcohol consumption prior to admission.

**Methods:**

The data were prospectively collected at admittance to an acute psychiatric department in the period January 2016 to June 2017. A blood sample for the analysis of PEth was available from 177 patients. We compared the PEth concentrations with the Alcohol Use Disorders Identification Test (AUDIT) scores during the hospital stay, and psychiatric diagnoses at discharge.

**Results:**

A total of 45.8% of the patients had a PEth concentration ≥ 0.03 μmol/L, indicating significant alcohol consumption. AUDIT scores consistent with unhealthy alcohol use were present in 51.7%. There was a significant positive correlation between PEth concentrations and AUDIT scores (r = 0.631, *p* < 0.001). PEth was above the detection limit of 0.03 μmol/L in 19% of those reporting an average daily intake of zero alcohol units per day during the last week before admission. PEth concentrations were significantly higher among those with an alcohol diagnosis than among those without such a diagnosis (0.82 μmol/L vs. 0.09 μmol/L, *p* = 0.001).

**Conclusion:**

PEth provides supplementary information on recent alcohol consumption in a psychiatric population and would be particularly helpful in patients unable or unwilling to give such information at admission.

**Supplementary Information:**

The online version contains supplementary material available at 10.1186/s12888-022-03934-y.

## Introduction

Alcohol use disorder (AUD) is prevalent in patients with mental illness, and is associated with a higher risk of suicide, somatic illness and shorter life-expectancy [1–4]. AUD is associated with intoxication, delirium, psychotic symptoms and alcohol withdrawal symptoms and can mimic several mental and medical conditions, which can complicate assessment and treatment [5]. AUD is also a risk factor for discontinuation of mental health treatment, reduced treatment effect and relapse of the psychiatric disorder [6]. In a Norwegian study, half of the patients admitted to an acute psychiatric inpatient department self-reported unhealthy alcohol use, and this group also had the highest risk of suicide and the shortest length of hospital stay [2]. Previous studies in psychiatric populations have confirmed an incidence of unhealthy alcohol use of about 50% [7]. Unhealthy alcohol use refers to all use of alcohol that increases the risk of harmful consequences or has already caused harmful consequences, and includes the term AUD [8, 9]. By definition, a diagnosis of AUD is fulfilled when the diagnostic criteria of DSM-5 is met, but to complicate terminology, ICD-10 operates with alcohol related disorder and includes several different terms from abuse and harmful use to dependence. In this article, we will therefore use the term AUD to describe alcohol use causing social, occupational, or health consequences, as a more severe condition than unhealthy alcohol use. Comorbid AUD can be difficult to identify and diagnose since a detailed information regarding alcohol use must be obtained, which can be challenging in acutely ill psychiatric patients. AUD is an underdiagnosed and thus also an undertreated disease in the psychiatric population [10]. Therefore, reliable and valid screening and assessment methods for detecting potentially unhealthy alcohol use is needed [11, 12].

The alcohol marker phosphatidylethanol (PEth) is produced only in the presence of ethanol in the body and is increasingly being used as a marker for alcohol intake and abstinence monitoring. Due to a detection window of up to several weeks after alcohol consumption, its application potential is broader than for other ethanol biomarkers [13]. PEth has been validated as a biological marker of alcohol use in several populations and settings [14–17]. In patients admitted to somatic emergency units, PEth has identified a high incidence of alcohol use, and has also proven to be useful in identifying individuals that underreport alcohol intake [18–21]. A study demonstrated that 15.6% of all patients admitted to a trauma unit who declared to be abstaining for the last 12 months had a PEth concentration indicating alcohol consumption during the recent weeks [21]. In critically ill patients, PEth has also been found to have strong discrimination qualities for alcohol misuse, with a receiver operator characteristics (ROC) area under the curve (AUC) of 0.948 (95% CI: 0.910, 0.956), compared to the Alcohol Use Disorders Identification Test C (AUDIT-C), consisting of three of the questions from the full AUDIT questionnaire [14].

We are not aware of any studies that have compared PEth and self-report assessment of alcohol use among patients admitted with acute psychiatric conditions. Hence, the aim of the present study was to investigate how PEth concentrations corresponds to AUDIT scores, self-reported alcohol intake and a diagnosis of alcohol disorder, and whether PEth can provide added value in a population of patients admitted to an acute psychiatric department.

## Materials and methods

### Population

**Fig. 1:**
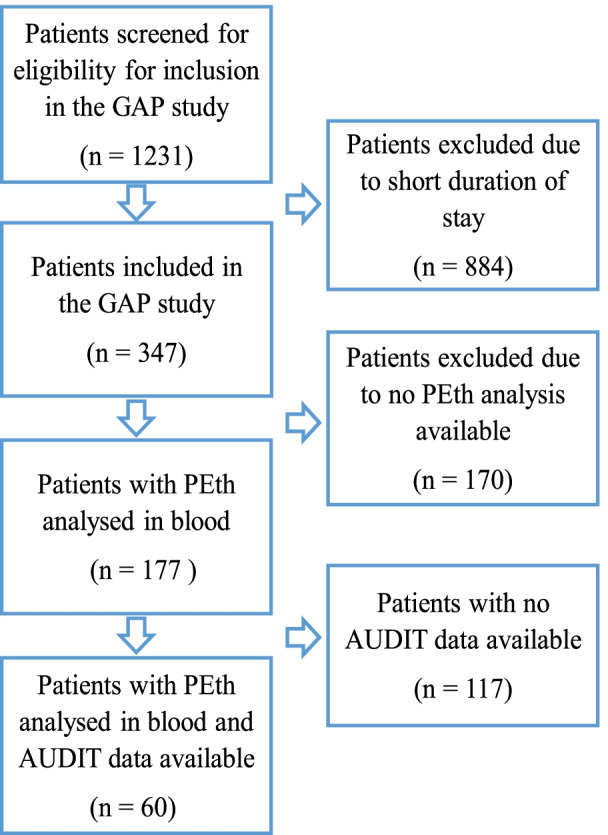
Flow diagram of patient inclusion into the data analysis. GAP study = Genetic and Affective Prediction Study, PEth = phosphatidylethanol, AUDIT = Alcohol Use Disorders Identification Test

Norwegian acute psychiatric services are publicly funded and available to all registered inhabitants in Norway. Department of Acute Psychiatry, St. Olav University Hospital, Trondheim, Norway, has a catchment area consisting of both urban and rural parts, covering about 310,000 residents [22]. All individuals ≥18 years living in this area in need of acute psychiatric services are referred to this department. Patients referred between January 2016 and June 2017 were invited to participate in the Genetic and Affective Prediction (GAP) study which aimed to explore predictors of affective impulsivity in psychiatric inpatients [23]. Altogether 1231 patients were admitted to the department during the study period, and among these, 347 were included in the GAP study. The reason for the high number of non-inclusion was that a large proportion of the patients had a very short duration of stay and did therefore not fulfil the criteria for being included [23]. In included patients who consented to blood sampling (*n* = 177), a PEth analysis was performed. Due to the late introduction of alcohol questionnaires in the study, AUDIT was performed on a selection of patients from whom a PEth concentration was available (*n* = 60) (Fig. 1).

### Examinations

All participants received an initial general psychiatric and physical examination by the physician on duty at admission. The research intake protocol together with demographic information were collected on arrival. The questionnaire regarding alcohol use was completed the first workday. The form was completed under the guidance of a trained nurse, and both the content and size of an alcohol unit were clarified. Blood samples were obtained the day after admission and the samples were kept refrigerated until analysis. Diagnoses according to the International Statistical Classification of Diseases, 10th Revision, (ICD-10) were set in joint meetings in the department [24]. At least two specialists, one of whom had examined the patient himself, were present at the meetings, who were blinded to the measured PEth concentration. The term “diagnosed with alcohol disorder” used in this article was defined as having an ICD-10 F10 diagnosis; “mental or behavioral disorder due to use of alcohol” [25]. This diagnosis largely corresponds to AUD as defined in Diagnostic and Statistical Manual of Mental Disorders, 5th edition (DSM-5) [26].

### Audit

AUDIT is a ten-item questionnaire with a total score from 0 to 40. It is a commonly used screening tool to asses alcohol use and to identify harmful alcohol consumption and dependence developed by the World Health Organization (WHO) [27, 28]. As mentioned in the introduction, we will hereafter use the term “unhealthy alcohol use” to encompass the different terms regarding AUDIT score > 8. Various cut-off scores for AUDIT has been applied, and in this study a score of zero to seven was classified as indication of low-risk alcohol consumption, a score from eight to 15 as unhealthy consumption, and a score of 16 or more as indication of alcohol dependence (moderate to severe AUD) [28]. In addition to AUDIT, seven questions regarding the amount of alcohol consumed during the last weeks prior to admission were included (the “extended AUDIT test”, Supplementary file 1). In Norway, one alcohol unit is defined as 12 g of pure alcohol corresponding to 33 cl beer (4.5%), 13 cl wine (12%) or 4 cl spirits (40%) [29], it is important to note that there is a variation in definitions of an alcohol unit between different countries [30].

### Analysis of PEth

Analyses of PEth in blood was performed at the Department of Clinical Pharmacology, St Olav University Hospital. The PEth 16:0/18:1 homologue was analysed with a validated ultra-performance liquid chromatography tandem mass spectrometry (UPLC®-MSMS) method described in detail previously [31].

There is no consensus on how to interpret a given PEth concentration, neither internationally nor in Norway [32]. However, Norwegian laboratories have a custom to interpret concentrations below 0.03 μmol/L as consistent with abstention or a very low level of alcohol consumption. PEth concentrations between 0.03 μmol/L and 0.30 μmol/L are interpreted as being consistent with moderate or significant alcohol consumption, whereas concentrations above 0.30 μmol/L are considered to be indicative of heavy alcohol consumption [33]. It is consensus that PEth concentrations reflect alcohol consumption in the two to four preceding weeks [34].

### Statistics

Data were analysed with SPSS version 27 (IBM Corp. Released 2020. IBM SPSS Statistics for Windows. Armonk, NY: IBM Corp).

Continuous data were compared by independent samples t-test or Mann-Whitney U-test, as appropriate. The Spearman rank correlation was used to assess the degree of association between two variables. ROC curves were created to evaluate the diagnostic value of PEth and AUC values with 95% confidence intervals (CIs) were calculated. Youden index (Youden’s J statistic) was calculated as J = sensitivity + specificity – 1. The index was defined for all points of the ROC curves, and its maximum value was used as a criterion for selecting optimum PEth cut-off concentrations.

## Results

A PEth analysis was performed in 177 participants, and an extended AUDIT test was performed on 60 of these (Fig. 1). The main characteristics of these populations are enlisted in Table 1.

**Table 1:** Patient characteristics in the complete study population (*n* = 177) and in the participants with AUDIT scores available (*n* = 60). The table presents the groups by age, gender, duration of stay, body mass index, diagnoses and phosphatidylethanol (PEth) concentrations

	Study population (***n*** = 177)	Patients with AUDIT scores (***n*** = 60)
Men n (%)	84 (47.5)	29 (48.3)
Women n (%)	93 (52.5)	31 (51.7)
Body mass index, kg/m^2^, mean (SD)	25.1 (5.0)	25.5 (4.8)
Age, years, mean (SD)	38.1 (16.0)	39.50 (16.7)
Men, age, mean (SD)	39.9 (16.9)	39.7 (17.6)
Women, age, mean (SD)	36.5 (15.0)	39.2 (16.0)
Duration of stay in days, mean (SD)	7.7 (5.7)	6.6 (4.3)
*Main diagnosis*		
F10-19 Mental and behavioral disorders due to psychoactive substance use, n (%)	23 (13)	7 (11.7)
F20-29 Schizophrenia, schizotypal and delusional disorders n, (%)	9 (5.1)	4 (6.7)
F30-39 Mood [affective] disorders, n (%)	59 (33.3)	23 (38.3)
F40-48 Neurotic, stress-related and somatoform disorders, n (%)	34 (19.2)	13 (21.7)
F50-59 Behavioral syndromes associated with physiological disturbances and physical factors, n (%)	1 (0.6)	0
F60-69 Disorders of adult personality and behavior, n (%)	26 (14.7)	8 (13.3)
Other disorders, n (%)	25 (14.1)	5 (8.3)
Alcohol diagnosis (F10.0-10.9), n (%)^a^	27 (15.3)	10 (16.7)
PEth concentration > 0.03 μmol/l, n (%)	81 (45.8)	31 (51.7)

^a^Includes 12 patients diagnosed with an alcohol disorder diagnosis (F10.0-F10.9) as a primary diagnosis, 13 with alcohol disorder as secondary diagnosis, and two patients with alcohol disorder both as a primary and a secondary diagnosis

### PEth concentrations

**Fig. 2:**
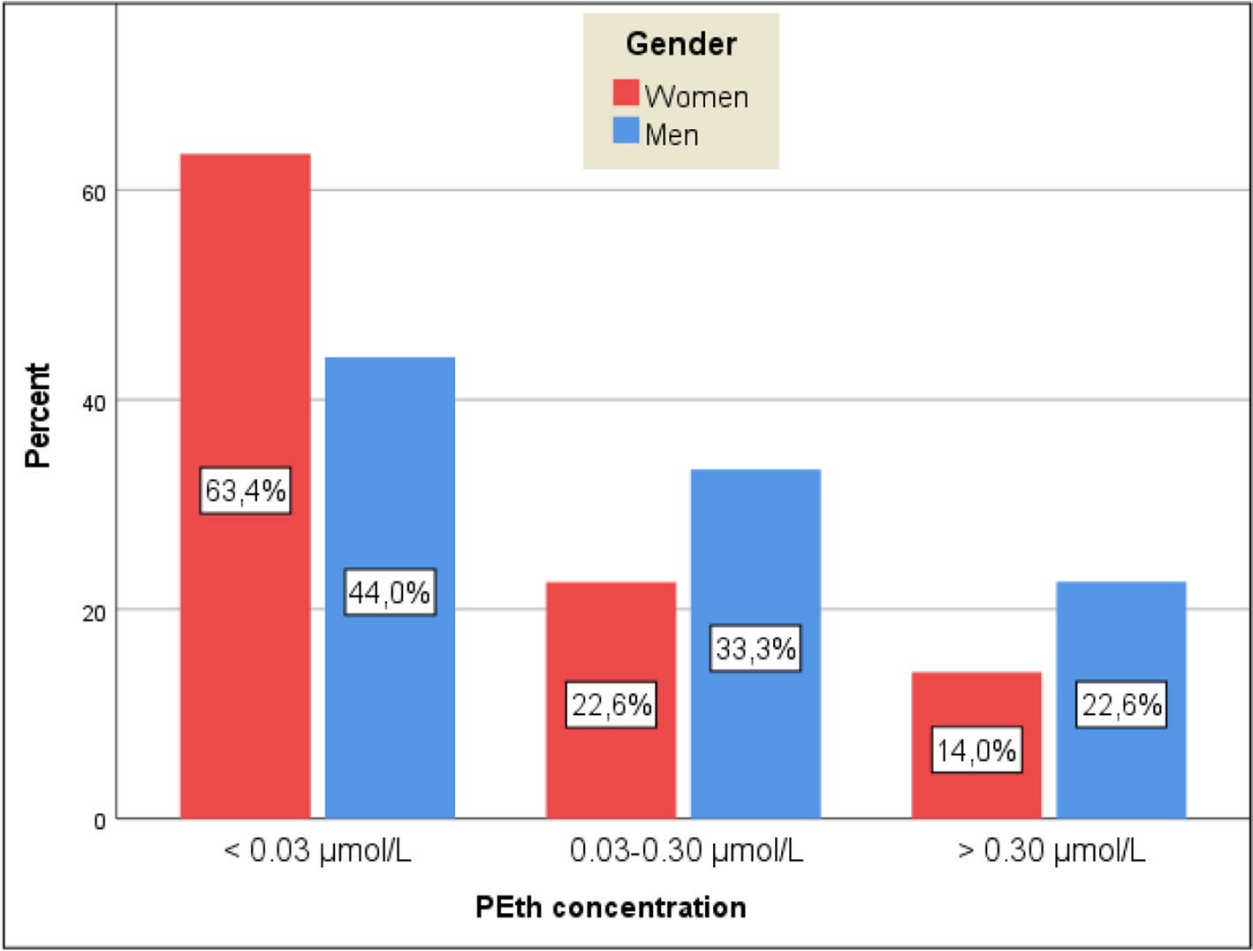
Distribution of phosphatidylethanol (PEth) concentrations in the study population

A total of 81 patients (45.8%) had PEth concentrations ≥0.03 μmol/L, whereas 32 (18.1%) had PEth concentrations ≥0.30 μmol/L. Consequently, 96 patients (54.2%) had PEth concentrations < 0.03 μmol/L. The distribution of PEth concentrations according to gender is presented in Fig. 2. Mean PEth concentrations were 0.21 μmol/L in the total population, 0.11 μmol/L among women and 0.31 μmol/L among men. A detailed description of PEth concentration by gender is presented in supplementary Table 1.

### PEth vs. AUDIT

**Fig. 3:**
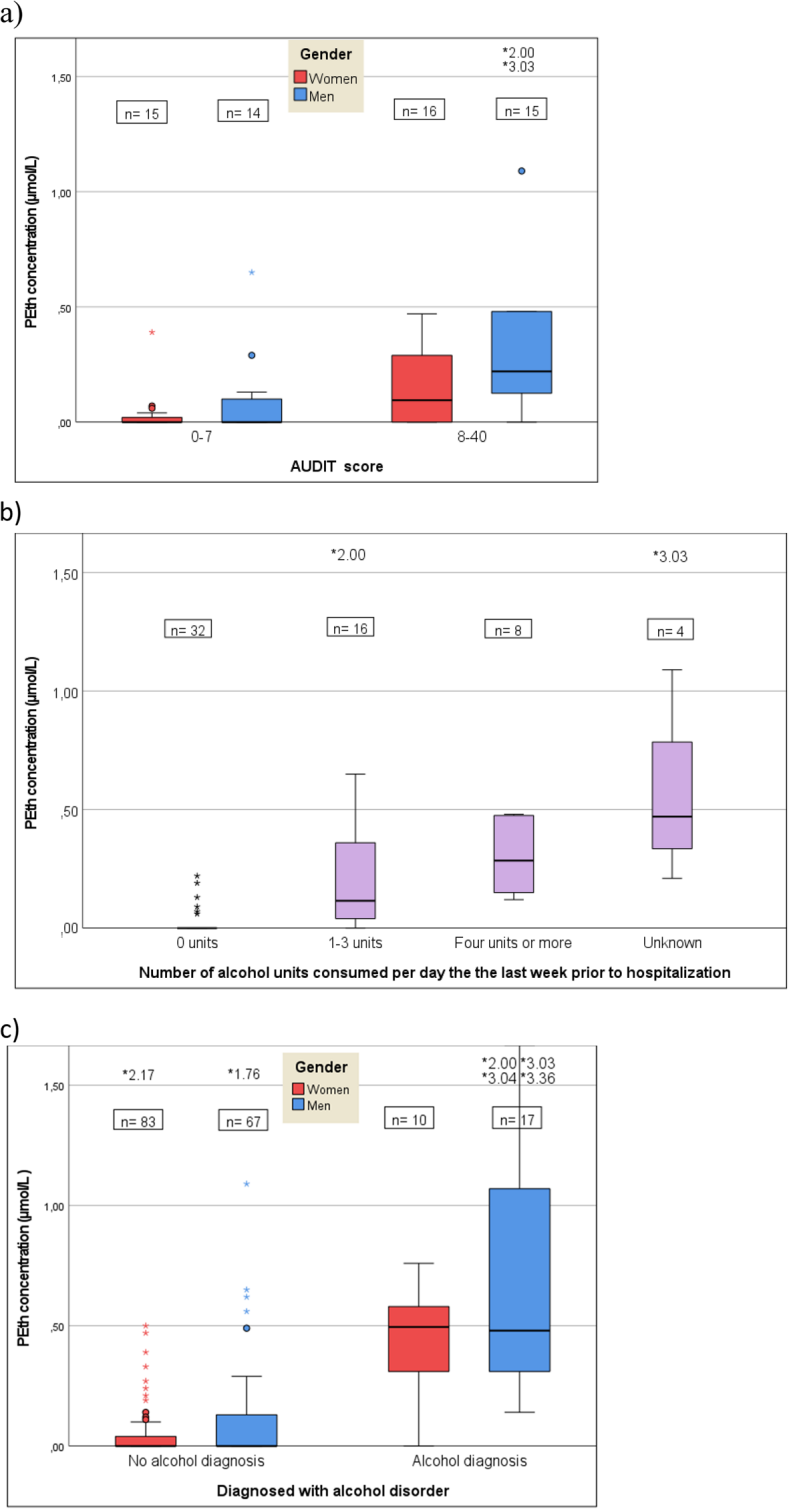
Boxplots of hosphatidylethanol (PEth) concentrations by: **a** Alcohol Use Disorders Identification Test (AUDIT) score (*n* = 60, **b** average daily alcohol consumption the last week prior to hospitalization (*n* = 60) and c) diagnosed with alcohol disorder (*n* = 177). Two male patients in figure **a**) and **b**) with PEth concentrations above 1.5 μmol/L are not shown to increase readability. One of these had a PEth concentration of 2.00 μmol/L, no alcohol diagnosis, an AUDIT score of 11 and reported drinking 1-3 alcohol units per day. The other had a PEth concentration of 3.03 μmol/L, an alcohol diagnosis, an AUDIT score of 14 and reported drinking ≥4 alcohol units per day. In figure **c**) six patients with PEth concentrations above 1.5 μmol/L are not shown. Boxes represent the first quartile and third quartile, the central line represents median, whiskers represent minimum and maximum levels excluding outliers. Circles represent outliers with values between 1.5 and 3 times the interquartile range, the asterisks represent outliers more than 3 times the interquartile range

Among those with available AUDIT scores, 31 (51.7%) had scores ≥8, i.e., consistent with unhealthy alcohol use (Fig. 3a). Thirteen patients (21.7%) had AUDIT scores ≥16 indicating alcohol dependence. There was a significant correlation between PEth concentrations and AUDIT scores (r = 0.631, *p* < 0.001). Mean PEth concentrations in those with AUDIT scores ≥8 and < 8 were 0.36 μmol/L (SD 0.63) and 0.06 μmol/L (SD 0.15), respectively (*p* = 0.001). Women with AUDIT scores ≥8 had a mean PEth concentration of 0.15 μmol/L (SD 0.17), while men in the same group had a mean PEth concentration of 0.58 μmol/L (SD 0.86) (*p* = 0.066). In the group with AUDIT scores < 8 women had a mean PEth concentration of 0.04 μmol/L (SD 0.10) and men 0.08 μmol/L (SD 0.18) (*p* = 0.78).

Eight of the 29 patients (27.6%) with AUDIT scores < 8 had PEth concentrations ≥0.03 μmol/L, including two (6.9%) with concentrations ≥0.30 μmol/L. The two patients having AUDIT scores < 8 and PEth concentrations ≥0.30 μmol/L, had PEth concentrations of 0.39 and 0.65 μmol/L, respectively.

### PEth vs. self-reported alcohol consumption

There was a significant correlation between PEth concentrations and self-reported alcohol consumption the previous week (r = 0.77, *p* < 0.001) (Fig. 3b). Among the 32 patients stating to consume an average of zero alcohol units per day during the previous week, six (18.8%) had PEth concentrations ≥0.03 μmol/L (range 0.06 to 0.22 μmol/L). Of those reporting intake of 1-3 alcohol units per day, 81% had PEth concentrations ≥0.03 μmol/L (0.04-2.0). All subjects reporting intake of 4 or more alcohol units per day had PEth concentrations ≥0.03 μmol/L (range 0.12 to 3.03 μmol/L).

### PEth vs. diagnosed with alcohol disorder

A total of 27 of the 177 patients (15.3%) received a main or secondary diagnosis of alcohol disorder (F10.0 - F10.9) during their hospital stay. PEth concentrations ≥0.3 μmol/L were found in 21 (77.8%) of these. Conversely, a diagnosis of alcohol disorder was assigned to 21 of the 32 patients (65.6%) with PEth concentrations ≥0.3 μmol/L. PEth concentrations were significantly higher among those diagnosed with an alcohol disorder than among those without such a diagnosis 0.82 μmol/L (SD 0.92) vs. 0.09 μmol/L (SD 0.27); *p* < 0.001) (Fig. 3c). Less women (10/93; 10.8%) than men (17/84; 20.2%) were diagnosed with alcohol disorder, and among them women had a mean PEth concentration of 0.45 μmol/L (SD 0.23) while men had a mean PEth concentration of 1.05 μmol/L (SD 1.10) (p < 0.001). In the group not diagnosed with any alcohol disorder, women had a mean PEth concentration of 0.07 μmol/L (SD 0.25), while men had a mean PEth concentration of 0.12 μmol/L (SD 0.28).

### ROC curve analysis

**Fig. 4:**
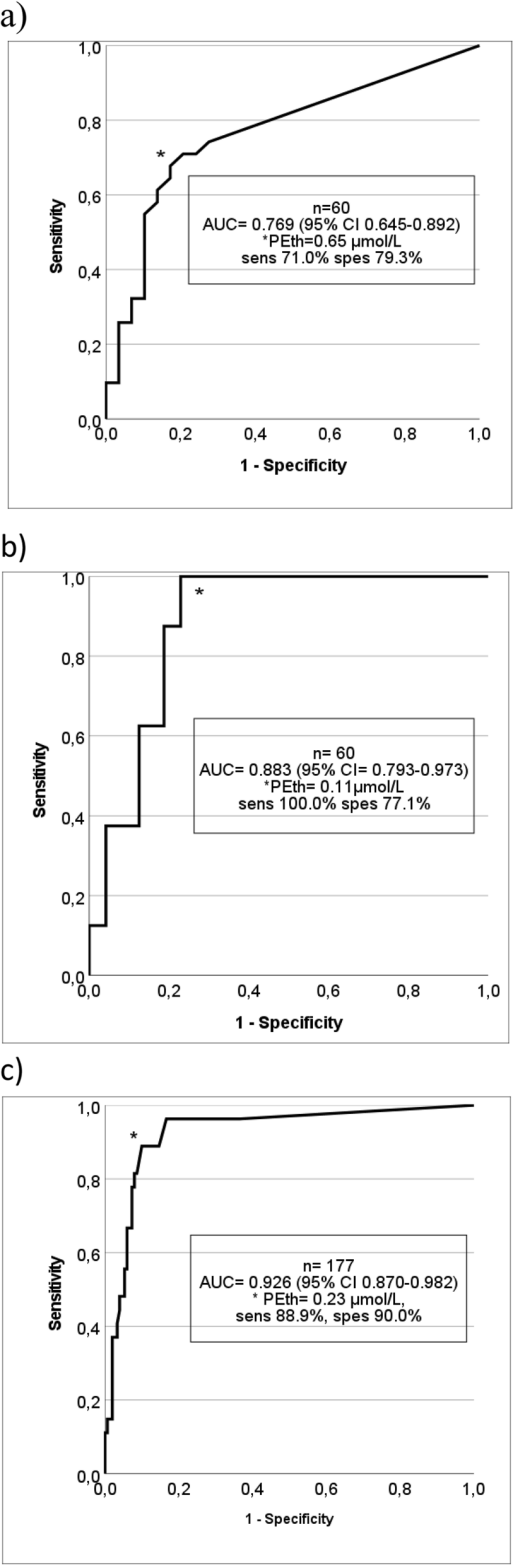
Receiver operating characteristics (ROC) curves of phosphatidylethanol (PEth) as a test for: **a** an AUDIT score of eight and above, **b** an average daily consumption of four or more alcohol units the last week, and **c** diagnosed with alcohol disorder. AUC = area under the ROC curve. Asterisks indicate the location of Youden’s index in each curve, with details found in the embedded frames

ROC curves for PEth as a marker for high AUDIT score, alcohol intake and having an alcohol disorder are shown in Fig. 4. AUC values of the ROC curves were 0.769 (95% CI 0.645-0.892) for having an AUDIT score ≥ 8, 0.883 (95% CI 0.793-0.973) for having consumed four or more alcohol units the last week, and 0.926 (95% CI: 0.870-0.982) for being diagnosed with an alcohol disorder shown in Fig. 4a), b) and c) respectively. The optimal cut point with Youden index and corresponding sensitivity, specificity and PEth concentration is also presented in Fig. 4. Due to the limited number of participants we did not perform gender-specific ROC analyses.

## Discussion

The main finding in the present study was the significant correlation between PEth concentrations and AUDIT scores, and between PEth concentrations and self-reported alcohol consumption. Patients that received an alcohol disorder diagnosis during their stay had significant higher PEth concentrations than those who did not. More than 50% of the participants reported unhealthy alcohol use according to the AUDIT score, and almost one of five among those reporting no intake of alcohol the last week tested positive for PEth, indicating significant alcohol consumption.

We decided to operate with a cut-off value for AUDIT of eight for both men and women. This is in accordance with the WHO guidelines from 2001 [28]. Lower cut-off values have been proposed for women, and also used in other studies, where cut-off values of five or six have been applied for women [35, 36].

For PEth, as mentioned earlier, there are no national or international guidelines with respect to clinical interpretation of its concentrations, but the guidelines used in Norway are common practice in clinical settings in several countries [32]. There is no indication that one should differentiate between men and women in the interpretation of PEth results, as no clear evidence for gender differences regarding formation or elimination of PEth has been found [37]. The findings in the present study that more men were positive for PEth and men had higher PEth concentrations, as seen in Fig. 1, are in line with what is known about alcohol and gender; women are less often current drinkers than men, and when women drink, they drink less than men [38, 39].

PEth concentrations and its clinical interpretation suggest that a consumption of two to four alcohol units several days a week is necessary to give a PEth concentration at or above 0.03 μmol/L, indicating significant or moderate consumption [32]. Heavy consumption, resulting in PEth concentrations > 0.30 μmol/L, is proposed to reflect a consumption of at least four alcohol units several days a week [32]. It is important to note that false positive PEth concentrations are not considered a clinical problem since PEth is formed only in the presence of alcohol.

A key aim of the present study was to compare PEth concentrations with AUDIT scores (although PEth reflects alcohol consumption in recent weeks whereas AUDIT applies to alcohol consumption during the last 12 months). One can assume that for most participants the drinking pattern during the previous 2-4 weeks would be the same as the last year, however the fact that the two tests reflect different time periods may explain that not all participants with an AUDIT score ≥ 8 had a PEth value that indicated heavy alcohol consumption and vice versa. Different time windows may also explain why some people had a positive PEth even though they reported no alcohol intake the last week prior to admission, as questions about alcohol consumption in the preceding weeks were not included. It can be assumed that alcohol intake last week also reflects average alcohol intake in previous weeks, but for future research that compares PEth with reported consumption, it would be wise to obtain information about alcohol intake the last month.

Another possible explanation for the discrepancy when comparing AUDIT scores ≥8 with PEth concentrations ≥0.30 μmol/L is that the applied cut-off for PEth may be too high. Afshar et al. recently found that a PEth cut-off of 0.04 μmol/L (25 ng/mL) was optimal for screening for unhealthy alcohol use when using AUDIT scores of ≥5 for females and ≥ 8 for males as the reference standard [40]. Also Gerbase et al. evaluated optimal cut-off values for PEth to identify unhealthy alcohol use and set it to 0.026 μmol/L, when using AUDIT-C as a reference [21]. These PEth cut-offs are far lower than what we regarded as indicative for unhealthy alcohol consumption in our study, but are more in line with our AUDIT results. Also in this setting, it seems appropriate to mention the ROC curves presented in Fig. 1, where a 100% sensitivity is seen when PEth is equal to 0.11 μmol/L.

No previous studies have been performed in psychiatric emergency patients using PEth as a marker of alcohol use. However, PEth has been evaluated to identify unhealthy alcohol consumption in several populations of somatic patients. A cross-sectional study of acutely medically ill patients in Oslo, Norway, showed that the prevalence of unhealthy alcohol use was 11.4% when assessed by PEth concentrations ≥0.3 μmol/L, compared to 21.1% when using the AUDIT-4 questionnaire [18]. In contrast, the prevalence of unhealthy alcohol use was higher in the present population, where PEth concentrations ≥0.3 μmol/L were seen in 18.1% and AUDIT scores ≥8 in 51.7% of the patients. It has already been shown that combining AUDIT and PEth can improve detection of alcohol use in medical settings [14, 21, 41]. In emergency room patients, 38.5% of those who declared abstinence in the last 12 months according to AUDIT had a positive PEth test [19]. It is therefore reasonable to suggest that PEth can provide important information when alcohol use is denied or underestimated, e.g. due to fear of sanctions, stigma or other social consequences, and also for those who are unable to complete a questionnaire or do not cooperate. In a psychiatric setting, the mental state of the patient can obviously complicate obtaining information, making PEth particularly beneficial.

There are some factors that are worth noting when comparing the results from the present study with other studies. First, the study population that had completed the extended AUDIT questionnaire was small (only 60 patients included), which made it particularly difficult to evaluate subgroups. Second, in Norway, patients with symptoms primarily due to substance abuse are admitted to specialized substance abuse facilities and only patients with dominating psychiatric conditions are admitted to acute psychiatric inpatient departments. Third, also to be noted is that short hospital stays are characteristic of patients with AUD [2] and that our study did not include patients with short stays (Fig. 1). This could potentially have underestimated the number of cases with AUD in the population under study. Fourth, in addition, we used the same AUDIT cut-off score for men and women, although a lower cut-off has been suggested for women [42]. If we had applied a cut-off of 6 for women, it would have increased the number of patients with an unhealthy pattern of alcohol use from 31 (51.7%) to 34 (56.7%). Therefore, several factors in this study may have underestimated the prevalence of unhealthy alcohol use in patients admitted with acute mental illness.

## Conclusion

We found a significant correlation between PEth concentrations, self-reported alcohol consumption and the presence of the diagnosis of alcohol disorder. More than 50% of patients admitted for acute mental care reported unhealthy alcohol use and 21.7% reported alcohol dependence. Such large numbers indicate that a high focus on alcohol screening is needed in acute psychiatric care to be able to give appropriate treatment. As almost one of five among those reporting no intake of alcohol the last week tested positive for PEth, a PEth analysis may provide important additional information on alcohol consumption. PEth would also be a useful tool in situations where patients are unable or unwilling to report their alcohol intake.

## Supplementary Information

**Additional file 1.**

**Additional file 2: Supplementary Table 1.** Phosphatidylethanol (PEth) concentrations in μmol/L in the complete study population, and in women and men shown separately.

## Data Availability

The dataset used during the current study are not publicly available due to restrictions that apply to the availability. Data are however available from the corresponding author on reasonable request.

## Abbreviations

AUC: Area under the time–concentration curve
AUD: Alcohol use disorder
AUDIT: Alcohol Use Disorders Identification Test
BMI: Body mass index
GAP: Genetic and Affective Prediction study
ICD: International Statistical Classification of Diseases
PEth: Phosphatidylethanol 16:0/18:1
ROC: Receiver operator characteristics
WHO: World Health Organization
