# Supplementary material for: Identification of unhealthy alcohol use by self-report and phosphatidylethanol (PEth) blood concentrations in an acute psychiatric department
Source: BMC Psychiatry. 2022 Apr 21;22:286. doi: 10.1186/s12888-022-03934-y (PMC9026645; doi:10.1186/s12888-022-03934-y)
Supplement: Supplementary file 1 — Additional file 1. [file 12888_2022_3934_MOESM1_ESM.docx]

**Alcohol Use Disorder Identification Test**

1. How often do you drink alcohol?

2. How many units of alcohol (a drink, a glass of wine or 1 small bottle of beer)

do you take on a "typical" drinking day?

3. How often do you drink six units of alcohol or more?

4. How often during the last year were you unable to stop drinking after you

had started?

5. How often during the last year did you fail to do things you should have done due to drinking?

6. How often do you start your day with alcohol?

7. How often during the last year have you felt guilty about drinking?

8. How often over the past year has it been impossible to remember what happened the night before due to drinking?

9. Have you or others been injured as a result of your drinking?

10. Have a relative, friend or doctor worried about your drinking, or suggested

that you should reduce?

**Additional questions**

11. How many units of alcohol (a drink, a glass of wine or 1 small bottle of beer) did you drink the last day before admission?

12. How many units of alcohol (a drink, a glass of wine or 1 small bottle of beer) did you drink the penultimate day prior to admission?

13. How was the consumption of alcohol in the last two days before admission

compared to regular, daily consumption last month?

14. How many units of alcohol (a drink, a glass of wine or 1 small bottle of beer) did you drink on average per day in the last week before admission?

15. (If 0 on question 6) How many units of alcohol (a drink, a glass of wine or 1 small bottle of beer) did you drink on average per day in the last month before admission?

16. When was the last alcohol intake?

(Enter the day and time as accurately as you can)

17. Has alcohol consumption in the last month been special in any way?
