# Supplementary material for: Identification of unhealthy alcohol use by self-report and phosphatidylethanol (PEth) blood concentrations in an acute psychiatric department
Source: BMC Psychiatry. 2022 Apr 21;22:286. doi: 10.1186/s12888-022-03934-y (PMC9026645; doi:10.1186/s12888-022-03934-y)
Supplement: Supplementary file 2 — Additional file 2: Supplementary Table 1. Phosphatidylethanol (PEth) concentrations in μmol/L in the complete study population, and in women and men shown separately. [file 12888_2022_3934_MOESM2_ESM.docx]

**Supplementary table 1**

Phosphatidylethanol (PEth) concentrations in µmol/L in the complete study population, and in women and men shown separately .

|  | **25th percentile** | **Median** | **75th percentile** | **95th percentile** | **Mean** |
| --- | --- | --- | --- | --- | --- |
| **Complete study population (n =177)** | <0.03 | <0.03 | 0.17 | 0.85 | 0.21 |
| Women (n = 93, 52.5%) | <0.03 | <0.03 | 0.09 | 0.57 | 0.11 |
| Men (n = 84, 47.5 %) | <0.03 | 0.08 | 0.28 | 1.94 | 0.31 |
